# Effects of sodium butyrate on growth performance, antioxidant status, inflammatory response and resistance to hypoxic stress in juvenile largemouth bass (*Micropterus salmoides*)

**DOI:** 10.3389/fimmu.2023.1265963

**Published:** 2023-11-03

**Authors:** Dongqiang Hou, Min Li, Peijia Li, Bing Chen, Wen Huang, Hui Guo, Junming Cao, Hongxia Zhao

**Affiliations:** ^1^ Collaborative Innovation Center of Aquatic Sciences, Guangdong Key Laboratory of Animal Breeding and Nutrition, Institute of Animal Science, Guangdong Academy of Agricultural Sciences, Guangzhou, China; ^2^ College of Fisheries, Guangdong Ocean University, Zhanjiang, China

**Keywords:** sodium butyrate, largemouth bass, antioxidant system, inflammatory response, hypoxic stress

## Abstract

The aim of this study was to investigate the effects of sodium butyrate (SB) supplementation on growth performance, antioxidant enzyme activities, inflammatory factors, and hypoxic stress in largemouth bass (*Micropterus salmoides*). Diets were supplemented with different doses of SB at 0 (SB0), 0.5 (SB1), 1.0 (SB2) and 2.0 (SB3) g/kg. The hypoxic stress experiment was performed after 56 days of culture. The results showed that compared with the SB0 group, the final body weight, weight gain rate and protein deposition rate of the SB3 group were significantly increased (*P*<0.05), while FCR was significantly decreased (*P*<0.05). The contents of dry matter, crude lipids, and ash in the SB2 group were significantly higher than those in the SB0 group (P<0.05). The urea level was significantly decreased (*P*<0.05), and the glucose content was significantly increased (*P*<0.05) in the SB supplement group. Compared with the SB0 group, the SB2 group had significant reductions in the levels of serum triglyceride, cholesterol, elevated-density lipoprotein cholesterol, and low-density lipoprotein (*P*<0.05), and significant reductions in the levels of liver alkaline phosphatase and malondialdehyde (*P*<0.05). The total antioxidant capacity of the SB1 group was higher than that of other groups (*P*<0.05). Compared with the SB0 group, the mRNA expression of *TLR*22, *MyD*88, *TGF-β*1, *IL*-1*β* and *IL*-8 in the SB2 group significantly decreased (*P*<0.05). The cumulative mortality rate was significantly decreased in the SB2 and SB3 groups in comparison with that in the SB0 group after three hours of hypoxic stress (*P*<0.05). In a 56-day feeding trial, SB enhanced largemouth bass growth by increasing antioxidant enzyme activity and inhibiting *TLR*22-*MyD*88 signaling, therefore increasing cumulative mortality from hypoxic stress in largemouth bass.

## 1 Introduction

Largemouth bass (*Micropterus salmoides*) is extremely popular in China because of its delicious meat, quick growth, and great economic value (1). With large-scale intensive farming, high stocking density, excessive feed, imbalanced dietary composition, exogenous pathogenic bacteria, and viruses may result in oxidative stress (2–4). Hypoxic stress and other environmental stress responses are common problems that threaten largemouth bass culture. Sustained prolonged-term stress will considerably affect the growth and energy metabolism of fish, making them more susceptible to infection by various pathogens, resulting in the continuous spread of fish diseases (5). At the same time, it will also weaken its immune function and disease resistance, and may even lead to death, which will cause serious damage to economic interests and ecological balance (6). In aquaculture, factors such as water eutrophication, elevated culture density and abnormal weather can lead to decreased dissolved oxygen concentration in water, increasing the risk of anoxic stress (7). Hypoxic stress leads to excessive accumulation of reactive oxygen species in fish, resulting in protein denaturation, lipid peroxidation, cell damage, and eventually apoptosis (8, 9). Oxidative stress can damage immune cells and trigger inflammatory response, which is the cause of numerous diseases (10, 11). The antioxidant system can prevent the generation of reactive oxygen species and avoid immune cell damage (12). When fish are confronted with hypoxic stress, the body resists the stress through a complex series of physiological and biochemical processes such as lowering metabolic rate, increasing aeration and anaerobic respiration, and increasing the oxygen affinity of hemoglobin (13, 14). Hypoxic conditions have been demonstrated to negatively affect growth performance and immunity in Nile tilapia (*Oreochromis niloticus*) (15). In mammals, hypoxia enhances the production of lactate and glucose, affecting nutrient metabolism (16). Chronic hypoxia activates anaerobic glycolysis, reduces glycogen and lactate concentrations in the hepatic, and increases the expression of phosphoenolpyruvate carboxykinase in juvenile European perch (*Linnaeus*) (17). Hypoxic stress limits the sustainability of largemouth bass farming and highlights the urgent need for solutions to this problem in the aquaculture industry.

Over the years, studies have found that organic acids and their salts positively affect animal nutrition and health (18). Short-chain fatty acids (SCFAs) including formic acid, acetic acid, and butyric acid (19), can regulate body metabolism through actions such as chelation with certain elements, modulation of intestinal acidity, and promotion of proliferation of gastrointestinal mucosal epithelial cells (20–22). Anaerobic bacteria produce butyric acid by digesting carbohydrates, which can provide energy for intestinal cells, promote intestinal cell differentiation and proliferation, and enhance the intestinal environment (23, 24). Sodium butyrate (SB), a chelated form of butyric acid, is a popular ingredient in animal feed for its improved stability and palatability (25). The solid form of SB provides better handling and storage properties, making it a practical addition to animal feed formulations (26). SB has emerged as a highly regarded additive in aquatic animal nutrition, with growing evidence supporting its effectiveness in improving growth performance and promoting intestinal health (27), such as Nile tilapia (25, 28, 29), hybrid grouper (*Epinephelus fuscoguttatus ♀ × E. lanceolatus ♂*) (19), largemouth bass (*Micropterus salmoides*) (30), turbot (*Scophthalmus maximus L.*) (31), yellow catfish (*Pelteobagrus fulvidraco*) (32), golden pompano (*Trachinotus ovatus*) (33). Several recent studies have found that SB has a role in improving immunity and antioxidant capacity (34). Transcription factor NF-E2-related factor 2 (Nrf2), as a protective transcription molecule, is considered to be the main regulator of cellular antioxidant defense response against environmental stress, and regulates the expression of cellular protection genes (35, 36). SB can regulate the activity of Nrf2 to promote the release of antioxidant enzymes and reduce cell apoptosis (37, 38). Sodium butyrate supplementation increased superoxide dismutase (SOD), catalase (CAT), and glutathione peroxidase (GSH-Px) activities in heat-stressed Nile tilapia (39). Dietary supplementation of 2.5 g/kg sodium butyrate increased the activity of lysozyme, decreased the expression of tumor necrosis factor α (*TNF-α*), and up-regulated the expression level of SOD in rainbow trout (Oncorhynchus mykiss) (40). In addition, previous studies in our laboratory have shown that an appropriate dose of sodium butyrate can enhance the resistance of juvenile yellow catfish to ammonia stress by improving antioxidant and immune responses (41). Based on these previous studies, SB offers a possible solution to hypoxic stress in aquatic animals. The effect of SB on improving antioxidants and immunity in aquatic animals, including largemouth bass, is unknown and deserves further exploration.

Largemouth bass, as a commercially valuable species, is one of the most economically significant freshwater fish in China with a production of more than 470,000 tons in 2019 (42, 43). It frequently experiences oxidative stress reactions such as hypoxia in pond culture, which threatens the health of the fish (43). The study aimed to address the health risks of oxidative stress in largemouth bass and to evaluate the effects of dietary sodium butyrate on blood metabolism, liver antioxidant status, immune response, and resistance to hypoxic stress in largemouth bass. This trial will elucidate the mechanism of action of sodium butyrate in protecting the health of largemouth bass. It will provide valuable insights to support the well-being of these aquatic organisms.

## 2 Materials and methods

All experimental procedures were performed in accordance with the Guide for Laboratory Animals of the Guangdong Academy of Agricultural Sciences, China. Animal use and care protocols were reviewed and approved by the Animal care and Use Committee of the Guangdong Academy of Agricultural Sciences (SC-GDAAS-2021-032).

### 2.1 Experimental diets

The formula and proximate composition of the experimental diet were shown in **Table 1**. Fish meal and soybean meal are the main proteins, and fish oil and soybean oil are the main lipids. In the study of yellow catfish supplemented with 0.25-2.0 g/kg sodium butyrate in the diet of our laboratory, it was found that the optimal supplemental amount of sodium butyrate was between 0.5-1.0 g/kg according to the growth, digestive enzyme activity and antioxidant indexes (32, 41). According to the results of previous experiments, the dosage of sodium butyrate in this experiment was determined to be 0.5-2.0 g/kg. Four isonitrogenous (50%) and isolipidic (9%) diets containing 0 (control, SB0), 0.5 g/kg (SB1), 1.0 g/kg (SB2), and 2.0 g/kg (SB3) SB were prepared. Eighty percent of the SB was purchased from Shanghai Yuanye Bio-Technology (Co., Ltd, China). All the ingredients were ground (AHZC1265 Hammer Mill, Buhler Machinery Co., LTD., Guangzhou, China) and, passed through a 60-mesh sieve, weighed accurately according to the formula. The blended feed ingredients were well mixed with lipid and water (30%) (AHML2000 Mixer, Buhler Machinery Co., LTD., Changzhou, China) before being structured into 2-mm pellet feed (pressure 1.83 MPa, SLX-80 Twin-screw Extruder, South China University of Technology Machinery Factory), dried at 55°C (HMO-205 Oven Dryer, Dongguan Haiming Electronic Technology Co., LTD., China), and kept at -20°C until use. The exact SB contents in the four diets were 0.0 g/kg, 0.38 g/kg, 0.75 g/kg, and 1.78 g/kg, which were measured by high-performance liquid chromatography (HPLC) (44).

**Table 1:** Formulation and proximate composition of the experimental diets (air-dry basis, %).

Ingredients	Contents (%)			
Fish meal (Peru, crude protein 67.7%) ^a^	35.00	35.00	35.00	35.00
Soy protein concentrate (crude protein 64.6%) ^a^	9.00	9.00	9.00	9.00
Blood meal ^a^	3.00	3.00	3.00	3.00
Shrimp shell meal ^a^	5.00	5.00	5.00	5.00
Soybean meal^a^	20.00	20.00	20.00	20.00
Cottonseed protein meal^a^	9.00	9.00	9.00	9.00
Tapioca meal^a^	9.00	8.95	8.90	8.80
Fish oil^a^	3.00	3.00	3.00	3.00
Soybean oil^a^	3.00	3.00	3.00	3.00
Vitamin premix^b^	1.00	1.00	1.00	1.00
Mineral premix^c^	1.00	1.00	1.00	1.00
Ca(H_2_PO4)_2_ ^a^	1.00	1.00	1.00	1.00
Choline chloride^a^	0.20	0.20	0.20	0.20
Sodium alginate^a^	0.80	0.80	0.80	0.80
Sodium butyrate	0.00	0.05	0.10	0.20
Total	100.00	100.00	100.00	100.00
Nutrients compositions of experimental diets (dry matter basis, %)^1^				
Crude protein	50.55			
Crude lipid	9.63			
Ash	12.44			
Moisture	3.73			

a Fishtech Fisheries Science & Technology Company, LTD, Institute of Animal Science, Guangdong Academy of Agricultural Sciences (Guangzhou, China).

b One kilogram of vitamin premix contained the following: VA 4,000,000 IU, VD_3_ 2,000,000 IU, VE 30 g, VK_3_ 10 _g_, VB_1_ 5 g, VB_2_ 15 g, VB_6_ 8 g, VB_12_ 0.02 _g_, nicotinic acid 40 g, calcium pantothenate 25 g, folic acid 2.5 g, inositol 150 g, biotin 0.08 g. Moisture ≤ 10%.

c One kilogram of mineral premix contained the following: MgSO_4_·H_2_O 12 g, KCl 90 g, Met-Cu 3 g, FeSO_4_·H_2_O 1 g, Ca (IO_3_)_2_ 0.06 g, Met-Co 0.16 g, ZnSO_4_·H_2_O 10 g, NaSeO_3_ 0.003 6 g. Moisture ≤ 10%.

^1^Nutrient composition is the actual measured value.

### 2.2 Feeding management

The Institute of Animal Science, Guangdong Academy of Agricultural Sciences (Guangzhou, China), conducted this experiment in an indoor recirculating aquaculture system. Largemouth bass was obtained from Guangzhou Jinglong Fishery (Guangzhou, China). Acclimate to the experimental settings for 14 days, during which the fish were fed the control food twice daily. After that, 480 healthy fish of similar weight (5.02 ± 0.01 g) were randomly assigned to 12 fiberglass tanks (300 liters) with 40 fish per tank in triplicate for each treatment. Fish were fed two times daily at 8:30 and 16:30 to apparent satiation. Half an hour after the end of each feeding, check the bottom of the cylinder for the presence of excess feed, if any, siphon the residual feed out. We collected any unconsumed feed to calculate overall consumption. The daily food intake of fish in each tank was recorded and used to calculate adjusted intake based on the previous day’s consumption. During the 56-day feeding trial, the water temperature was between 28 and 30°C, pH 7.3–7.7, ammonia ≤ 0.02 mg/L, nitrite ≤ 0.2 mg/L, and dissolved oxygen ≥ 6.0 mg/L, and the photoperiod regime was 12 h light and 12 h dark.

### 2.3 Sample collections and analysis

Fish were fasted for 24 hours after the terminal of the feeding trial and anesthetized with 40 mg/L MS-222 (Sigma, USA) for weighing and counting. The number and weight of fish in each tank were recorded to determine the final body weight (FBW), weight gain rate (WGR) and feed conversion ratio (FCR). The condition factor (CF) was determined by weighing and measuring the body length of six fish from each tank. Three fish were picked at random from each tank and held at -20°C for analysis of the approximate makeup of their whole bodies. The protein deposition rate (PDR) was calculated based on body composition and feed intake. Blood samples were collected from the tail vein of the fish using a 1 mL syringe and processed for coagulation at − 4°C. We centrifuged the collected blood samples at 3500 rpm for ten min in a centrifuge and then extracted the serum and stored it at − 80°C for subsequent examination. Fish intact livers were placed in tubes, temporarily frozen in liquid nitrogen, and stored at − 80°C after the essay for quantitative real-time PCR of antioxidant enzyme activity and expression of inflammation-related genes.

Crude protein, crude lipid and ash content in fish feed and whole fish were determined according to the Association of Official Analytical Chemists (45). Dry matter (DM) was measured by drying the samples to a constant weight at 105°C (AOAC, 1999; #930.15). Crude protein (N × 6.25) was determined by semi-automatic Kjeldahl nitrogen determination system after acid digestion (AOAC, 1999; #2001.11). Crude lipid content was determined by weight after lipid extraction by Soxhlet extraction using ether (36680 analyzer, Buchi, Switzerland) (AOAC, 1999; #920.39). Ash content was measured after six h combustion in a muffle furnace at 550°C (AOAC, 1999; #942.05). Serum metabolic parameters such as glucose (GLU), triglycerides (TG), urea nitrogen (UREA), cholesterol (CHO), high-density lipoprotein cholesterol (HDL), and low-density lipoprotein cholesterol (LDL) were examined using an automated chemical analyzer (Hitachi 7180, Tokyo, Japan).

Hepatic samples were homogenized and centrifuged at 2500 rpm for 15 minutes at 4°C according to the instructions of the kit, and the supernatant was separated for analysis. Superoxide dismutase (SOD), catalase (CAT), total antioxidant capacity (T-AOC), glutathione peroxidase (GSH-Px), alkaline phosphatase (AKP), and malondialdehyde (MDA) were determined using commercial kits (Nanjing Jiancheng Institute of Biological Engineering, Nanjing, China) in accordance with the manufacturer’s instructions (46).

### 2.4 Hypoxia stress experiment

At the end of the 56-day experiment, 16 fish from each tank were selected for the hypoxic stress test. The amount of water in each tank was maintained at approximately 40 L, the water surface was covered with a transparent plastic film, and the water circulation of the farming system was turned off (35). The dissolved oxygen meter was inserted into the gas cylinder and the cumulative number of dissolved oxygen and fish deaths was recorded every half hour. Dissolved oxygen in the water was maintained at 0 mg/L after 2 h of the test. At 3 h, fish mortality in the SB0 group reached 50%, at which point the experiment was stopped and the number of dead fish in each test pool was recorded. Fish were considered dead when there was no respiratory activity and no response to mechanical stimuli.

The cumulative mortality rate (CMR) was calculated as follows: CMR (%) = 100×the number of dead fish after the stress test/the number of fish before the stress test.

### 2.5 RNA extraction and real-time quantitative PCR analysis

The hepatic’s total RNA was extracted using the Trizol (Invitrogen, USA) method, and its quality and quantity were evaluated using 1% agarose gel electrophoresis and spectrophotometric analysis (A 260:280 nm ratio). The RNA was subsequently transcribed into cDNA using the HiScript^®^ III RT SuperMix kit (Vazyme, Nanjing, China). Primer sets were designed to quantify gene mRNA levels based on the sequences of immune and antioxidant-related genes of largemouth bass in GenBank (**Table 2**). The selected primers were synthesized by Shanghai Bioengineering Technology Co., Ltd., and real-time quantitative PCR (RT-qPCR) was performed on an ABI 7500 real-time PCR machine (Applied Biosystems, USA). β-actin was used as a non-regulatory reference gene in largemouth bass studies. Gene sequence amplification efficiency was close to 100%. In our present study, β-actin gene expression in the intestinal was also stable and not significantly affected by dietary SB. For all reactions, mRNA expression samples were assayed in triplicate, and gene expression levels were finally analyzed by the 2^−ΔΔCT^ method.

**Table 2:** Primer sequences for RT-qPCR analysis.

Genes^1^	Sequence Information		
	Forward primer (5′-3′)	Reverse primer (5′-3′)	GenBank
*β-actin*	GGACACGGAAAGGATTGACAG	CGGAGTCTCGTTCGTTATCGG	XM_038695351.1
*IL-*1*β*	CGTGACTGACAGCAAAAAGAGG	GATGCCCAGAGCCACAGTTC	XM_038696252.1
*IL-*8	CGTTGAACAGACTGGGAGAGATG	AGTGGGATGGCTTCATTATCTTGT	XM_038704088.1
*IL-*10	CGGCACAGAAATCCCAGAGC	CAGCAGGCTCACAAAATAAACATCT	XM_038696252.1
*TNF-α*	CTTCGTCTACAGCCAGGCATCG	TTTGGCACACCGACCTCACC	XM_038710731.1
*TGF-β*1	GCTCAAAGAGAGCGAGGATG	TCCTCTACCATTCGCAATCC	XM_038693206.1
*TLR*22	TCGCTGTTCACCAATCTG	TAGTTCTCCTCTCCATCTGT	MN807054.1
*MyD*88	CTCAACCCCAAGAACACA	CGAAGATCCTCCACAATG	XM_038728827.1
*IGF-*1	CTTCAAGAGTGCGATGTGC	GCCATAGCCTGTTGGTTTACTG	DQ666526
*GH*	CCCCCAAACTGTCAGAACT	ACATTTCGCTACCGTCAGG	DQ666528

The cDNA sequence of the target gene from NCBI (National Center for Biotechnology Information) was used, and the primer sequence was designed using primer 6, and then synthesized at Shanghai Biotechnology Co.

^1^IL-1β, interleukin 1β; IL-8, interleukin 8; IL1, interleukin 10; TNF-α, tumor necrosis factor α; TGF-β1, transforming growth factor β1; TLR22, toll-like receptor 22; MyD88, myeloid differentiation primary response gene 88; IGF-1, insulin-like growth factor-1; GH, growth hormone.

### 2.6 Calculation formula


WGR (%)=100×[final body weight (g) - initial body weight (g)] / initial body weight (g);

FCR= feed intake (g) / weight gain (g);

$$\begin{array}{l} \text{PDR }(\%)=100\times [\text{final body weight }(\text{g})\;\times \text{ final fish protein content }(\%)\\ \;-\text{initial body weight }(\text{g})\text{ × initial fish protein content }(\%)]\\ \;/\;\left[\text{feed intake }(\text{g})\text{ × feed protein content }(\%\right)]; \end{array}$$

hepatosomatic index(HSI, %) = 100 × hepatic weight (g)/ body weight (g);

visceralosomatic index (VSI, %) = 100 × viscera weight (g) / body weight (g);

$$\text{CF }({\text{g/cm}}^{3})= 100 \times live weight(\text{g})\text{ / body length }({\text{cm}}^{3});$$

### 2.7 Statistical analysis

All data analyzes were performed in the statistical software SPSS 25.0 (Chicago, USA) and reported as mean ± SEM. To test for changes in growth performance, enzyme activity, and gene mRNA expression among groups, data were processed as one-way ANOVA with both linear and quadratic significance tests followed by Duncan’s test to determine significant differences between treatments, with *P*<0.05 considered significant and 0.05≤*P*<0.10 considered trend effects. The final graph of this experiment was created using the GraphPad Prism 5 software (GraphPad Software U.S.A).

## 3 Results

### 3.1 Growth performance

**Table 3** presents information about the growth performance of experimental fish. Fish feed intake (FI) increased significantly with SB supplementation, and at the same time, FI decreased linearly and quadratic (P<0.05). The FBW and WGR of the basal diet and SB3-supplemented fish presented the lowest and highest values, respectively (*P*<0.05); that is, all experimental diets had higher FBW and WGR indices than the control diet. In addition, all diets containing the supplement reduced FCR, with SB2 and SB3 being significantly lower than the control (*P*<0.05). The SB3 group had a substantially greater PDR than the SB0 group (*P*<0.05). FBW, WGR and PDR increased linearly with the increase of SB (*P*<0.05). On the contrary, FCR decreased linearly with the increase of SB (*P*<0.05). The SB3 group had significantly lower hepatosomatic index (HSI) than the other groups (*P*<0.05). The visceralosomatic index (VSI) of largemouth bass-fed SB was not significantly different from that of the SB0 group (*P*>0.05). Compared to the SB0 group, dietary supplementation with SB showed no impact on CF in largemouth bass (*P*>0.05).

**Table 3:** Growth performance of largemouth bass fed a diet supplemented with sodium butyrate for 8 weeks.

Item	Dietary SB^1^ supplementation				SEM^2^	*P*-value	Polynomial contrasts	
	SB0	SB1	SB2	SB3			Linear	Quadratic
FI (g)	2196.83^b^	2336.30^a^	2317.17^a^	2326.80^a^	38.894	0.022	0.017	0.046
FBW (g)	50.55^b^	51.91^ab^	52.39^ab^	55.08^a^	1.424	0.067	0.014	0.530
WGR^3^ (%)	906.72^b^	934.55^ab^	944.21^ab^	998.07^a^	28.360	0.064	0.013	0.535
FCR^4^	1.25^a^	1.24^a^	1.22^b^	1.17^c^	0.007	< 0.001	0.017	0.355
PDR^5^ (%)	26.17^b^	26.22^b^	26.83^ab^	27.99^a^	0.570	0.041	0.010	0.208
HSI^6^ (%)	0.82^a^	0.87^a^	0.81^a^	0.71^b^	0.038	0.019	0.012	0.025
VSI^7^ (%)	5.95^ab^	7.04^a^	6.16^ab^	5.44^b^	0.565	0.108	0.215	0.052
CF^8^ (g/cm^3^)	2.02^ab^	1.76^b^	1.94^ab^	2.20^a^	0.172	0.152	0.215	0.063

FI, sum of daily feed intake (g); IBW, initial body weight (g): 5.02 ± 0.01; FBW, final body weight (g); WGR, weight gain rate (%); FCR, feed conversion ratio; PDR, protein deposition rate (%); HSI, hepatosomatic index (%); VSI, visceralosomatic index (%); CF, condition factor (g/cm^3^).

^1^SB, sodium butyrate. SB0, basal diet; SB1, basal diet supplemented with 0.5 g/kg of sodium butyrate; SB2, basal diet supplemented with 1.0 g/kg of sodium butyrate; SB3, basal diet supplemented with 2.0 g/kg of sodium butyrate.

^2^SEM, Standard error of the mean.

^3^WGR (%) = 100 × [final body weight (g) - initial body weight (g)]/initial body weight (g);

^4^FCR = feed intake (g)/weight gain (g);

^5^PDR (%) = 100 × [final body weight (g) × final fish protein content (%) − initial body weight (g) × initial fish protein content (%)]/[feed intake (g) × feed protein content (%)];

^6^HSI (%) = 100 × hepatic weight (g)/body weight (g);

^7^VSI (%) = 100 × viscera weight (g)/body weight (g);

^8^CF (g/cm^3^) = 100 × live weight (g)/body length (cm^3^);

^a,b,c^Bars without common superscripts indicate significant differences (*P<* 0.05).

### 3.2 Whole body composition

The effects of SB feeding on body composition of largemouth bass after 56 days are shown in **Table 4**. Compared with SB0 group, the contents of dry matter and crude lipid in SB2 group were significantly increased (*P*<0.05). Dry matter increased linearly and quadratic (*P*<0.05), and crude lipid increased linearly (*P*<0.05). Ash content in SB1 and SB2 groups was significantly increased (*P*<0.05). There was no significant difference in crude protein and water content among all groups (*P*>0.05). However, crude protein showed a secondary increase trend (*P*=0.077, 0.05<*P*<0.1).

**Table 4:** Effects of feeding supplemented Sodium butyrate diet for 56 days on body composition of largemouth bass (% wet weight).

Item^3^	Dietary SB^1^ supplementation				SEM^2^	*P*-value	Polynomial contrasts	
	SB0	SB1	SB2	SB3			Linear	Quadratic
Dry matter	27.71^b^	28.54^ab^	29.24^a^	28.79^ab^	0.455	0.052	0.026	0.081
Crude protein	15.99	16.65	16.59	16.39	0.300	0.199	0.270	0.077
Crude lipid	6.11^b^	6.54^ab^	7.15^a^	6.89^ab^	0.344	0.073	0.026	0.195
Ash	4.01^b^	4.22^a^	4.22^a^	4.13^ab^	0.067	0.047	0.162	0.014
Moisture	0.76	0.85	0.86	0.96	0.109	0.430	0.129	0.990

^1^SB, sodium butyrate. SB0, basal diet; SB1, basal diet supplemented with 0.5 g/kg of sodium butyrate; SB2, basal diet supplemented with 1.0 g/kg of sodium butyrate; SB3, basal diet supplemented with 2.0 g/kg of sodium butyrate.

^2^SEM: Standard error of the mean.

^3^Nutrient composition is the actual measured value.

^a,b,c^Bars without common superscripts indicate significant differences (*P<* 0.05).

### 3.3 Serum biochemistry

The effects of SB on serum biochemistry of largemouth bass are summarized in **Table 5**. Compared with the SB0 group, fish fed the SB diet had lower urea levels with a linear and quadratic decrease (*P*<0.05), while GLU was significantly higher with a linear and quadratic increase (*P*<0.05). The levels of TG, CHO, HDL and LDL of fish fed SB2 diet were lower than those of fish fed SB0 diet (*P*<0.05). There was a linear decrease in TG, HDL, and LDL, and a linear and quadratic decrease in CHO. Compared with the SB0 group, the AKP level in the SB2 group was significantly decreased, showing a linear and quadratic decrease (*P*<0.05).

**Table 5:** Serum biochemical correlation indices of largemouth bass fed dietary supplementation with different levels of sodium butyrate diets for 56 days.

Item^3^	Dietary SB^1^ supplementation				SEM^2^	*P*-value	Polynomial contrasts	
	SB0	SB1	SB2	SB3			Linear	Quadratic
GLU (mmol/L)	4.23^b^	7.20^a^	7.35^a^	7.67^a^	1.263	0.003	0.001	0.016
UREA (mg/dl)	7.48^a^	5.35^b^	4.97^b^	4.69^b^	0.459	0.001	< 0.001	0.022
TG (mmol/L)	2.48^a^	2.23^ab^	1.84^b^	2.02^ab^	0.226	0.096	0.039	0.206
CHO (mmol/L)	6.31^a^	6.07^a^	5.29^b^	5.65^b^	0.148	0.005	0.003	0.034
HDL (mmol/L)	2.99^a^	2.93^ab^	2.56^b^	2.75^ab^	0.174	0.127	0.076	0.325
LDL (mmol/L)	1.19^a^	1.08^ab^	0.84^c^	0.99^bc^	0.077	0.023	0.014	0.083
AKP (U/L)	83.54^a^	67.21^ab^	54.43^b^	61.54^b^	7.151	0.019	0.008	0.049

^1^SB, sodium butyrate. SB0, basal diet; SB1, basal diet supplemented with 0.5 g/kg of sodium butyrate; SB2, basal diet supplemented with 1.0 g/kg of sodium butyrate; SB3, basal diet supplemented with 2.0 g/kg of sodium butyrate.

^2^SEM, Standard error of the mean.

^3^GLU, glucose; TG, triglycerides; UREA, urea nitrogen; CHO, cholesterol, HDL, high-density lipoprotein cholesterol; LDL, low-density lipoprotein cholesterol. AKP, alkaline phosphatase.

^a,b,c^Bars without common superscripts indicate significant differences (*P<* 0.05).

### 3.4 Hepatic antioxidant enzyme activities

The effects of dietary SB on AKP, CAT, MAD, TAOC, SOD, and GSH-Px in the liver are shown in **Table 6**. MDA in SB supplementation group was significantly lower than that in SB0 group, showing a linear and quadratic decrease (*P*<0.05). The T-AOC of fish fed SB1 was higher than that of other groups (*P*<0.05). The levels of AKP, CAT, SOD and GSH-Px in each experimental group were similar (*P*>0.05).

**Table 6:** Antioxidant enzyme related indices of largemouth bass fed dietary supplementation with different levels of sodium butyrate diets for 56 days.

Item^3^	Dietary SB^1^ supplementation				SEM^2^	*P*-value	Polynomial contrasts	
	SB0	SB1	SB2	SB3			Linear	Quadratic
AKP	34.95	37.49	35.31	38.17	3.950	0.808	0.566	0.957
CAT	179.29	191.35	191.62	171.98	11.825	0.396	0.570	0.133
MDA	5.20^a^	3.94^b^	2.28^d^	3.10^c^	0.231	< 0.001	< 0.001	0.001
T-AOC	0.57^b^	1.47^a^	0.42^b^	0.39^b^	0.199	0.004	0.073	0.006
SOD	202.37	182.65	185.17	182.86	26.116	0.870	0.576	0.654
GSH-Px	456.39^c^	302.95^b^	341.97^b^	552.41^a^	70.112	0.072	0.214	0.021

^1^SB, sodium butyrate. SB0, basal diet; SB1, basal diet supplemented with 0.5 g/kg of sodium butyrate; SB2, basal diet supplemented with 1.0 g/kg of sodium butyrate; SB3, basal diet supplemented with 2.0 g/kg of sodium butyrate.

^2^SEM, Standard error of the mean.

^3^SOD, superoxide dismutase; CAT, catalase; T-AOC, total antioxidant capacity; GSH-Px, glutathione peroxidase; AKP, alkaline phosphatase; MDA, malondialdehyde.

^a,b,c^Bars without common superscripts indicate significant differences (*P<* 0.05).

### 3.5 Gene expression

#### 3.5.1 Hepatic signaling pathway related gene expression

The effects of dietary SB on liver immune and growth-related genes are shown in **Table 7**. The mRNA expression of *TLR*22 and *MyD*88 in the liver of juvenile largemouth bass fed with SB1, SB2 and SB3 diets was significantly lower than that in the SB0 group, with linear and quadratic reductions (*P*<0.05). The mRNA expression of *TLR*22 in the liver of the SB2 group was significantly lower than that in the SB0 group (*P*<0.05). Compared with the control group, the expression of *TGF-β*1 mRNA decreased linearly with the increase of SB supplementation (*P*<0.05).

**Table 7:** Expression of inflammatory factor in largemouth bass fed diets supplemented with different levels of sodium butyrate for 56 days.

Item^3^	Dietary SB^1^ supplementation				SEM^2^	*P*-value	Polynomial contrasts	
	SB0	SB1	SB2	SB3			Linear	Quadratic
*TLR*22	1.16^a^	0.97^b^	0.64^c^	0.71^c^	0.068	0.003	0.001	0.066
*MyD*88	1.25^a^	0.75^b^	0.44^b^	0.71^b^	0.127	0.007	0.006	0.006
*TGF-β*1	1.01^a^	0.92^a^	0.50^b^	0.39^b^	0.175	0.018	0.003	0.903
*IL-*1*β*	1.01^a^	0.72^bc^	0.51^c^	0.78^ab^	0.099	0.014	0.023	0.008
*IL-*8	2.24^a^	1.94^a^	0.83^b^	1.067^b^	0.173	0.003	0.001	0.094
*IL-*10	1.10	1.02	0.80	0.77	0.272	0.559	0.189	0.901
*TNF-α*	1.00	1.00	0.73	0.98	0.104	0.162	0.445	0.234
*IGF-*1	1.02	1.55	1.68	1.72	0.810	0.814	0.412	0.680
*GH*	1.01	1.31	1.57	1.78	0.491	0.477	0.903	0.137

^1^SB, sodium butyrate. SB0, basal diet; SB1, basal diet supplemented with 0.5 g/kg of sodium butyrate; SB2, basal diet supplemented with 1.0 g/kg of sodium butyrate; SB3, basal diet supplemented with 2.0 g/kg of sodium butyrate.

^2^SEM: Standard error of the mean.

^3^IL-1β, interleukin 1β; IL-8, interleukin 8; IL10, interleukin 10; TNF-α, tumor necrosis factor α; TGF-β1, transforming growth factor β1; TLR22, toll-like receptor 22; MyD88, myeloid differentiation primary response gene 88; IGF-1, insulin-like growth factor-1; GH, growth hormone.

^a,b,c^ Bars with different superscripts represent significant difference (*P*<0.05). β-Actin expression was used as an internal control for real-time PCR.

#### 3.5.2 Hepatic inflammation-related gene expression

In the diet supplemented with SB, *IL-*1*β* was linearly and quadratic decreased (*P*<0.05), and the relative expression of *IL-*8 was linearly decreased (*P*<0.05). The expression levels of *TNF-α* and *IL-*10 in fish diets supplemented with SB tended to decrease, but there was no statistical significance between groups (*P*>0.05).

#### 3.5.3 Hepatic growth-related gene expression

Dietary SB supplementation increased *GH* and *IGF-1* mRNA expression in the liver of largemouth bass, but none of the groups showed statistical significance (*P*>0.05).

### 3.6 hypoxic stress

**Figure 1:**
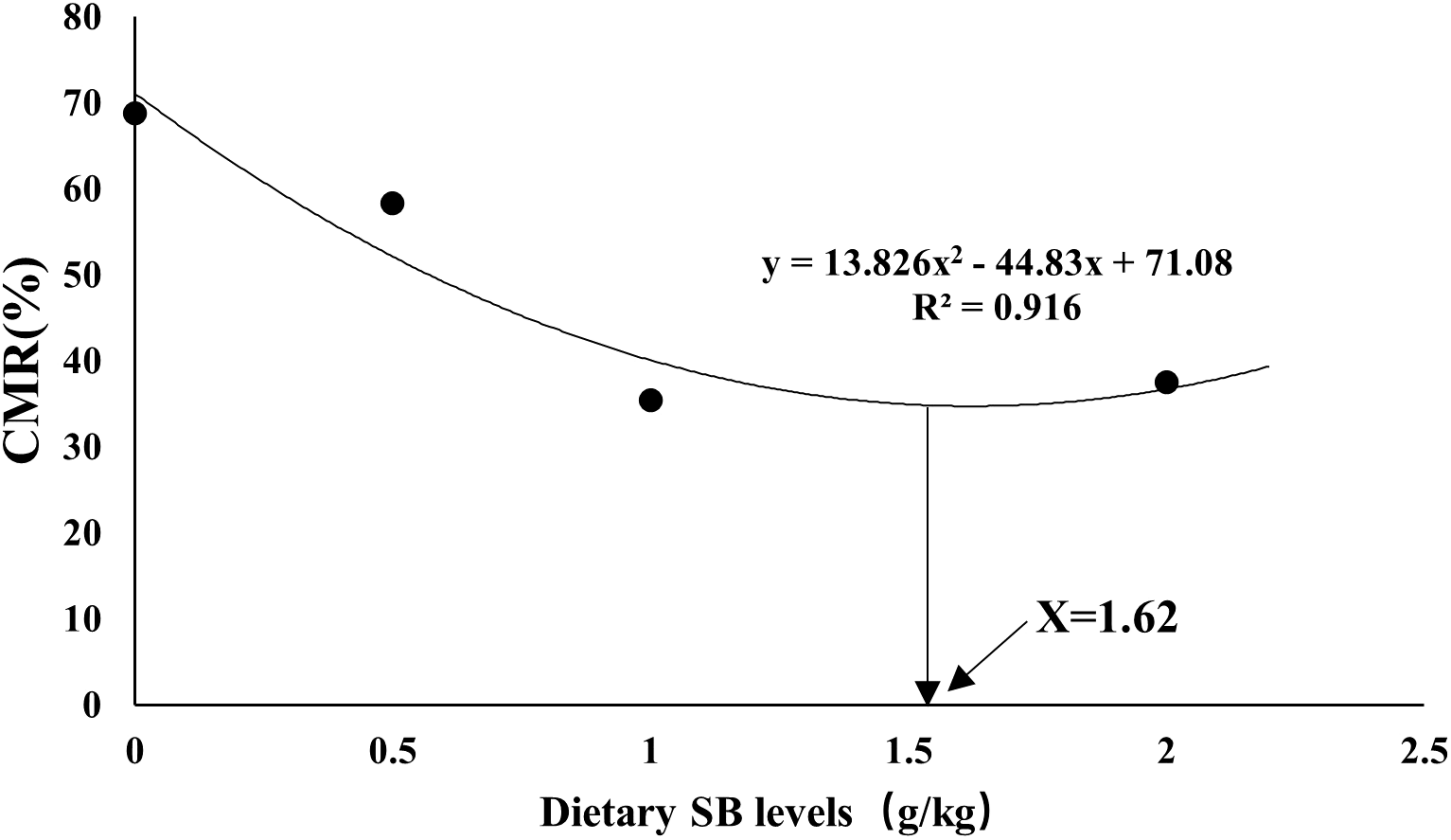
Quadratic regression analysis of cumulative mortality (CMR, %) and dietary SB level of largemouth sea bass after 56 days of hypoxia stress.

**Table 8** shows the cumulative mortality of largemouth bass juvenile exposed to hypoxic stress for 3 hours. After 2 h of hypoxic stress, the cumulative mortality in SB2 and SB3 groups was significantly lower than that in SB0 group (*P*<0.05). After 3 hours of hypoxic stress, the cumulative mortality rates in the SB0, SB1, SB2 and SB3 groups were 68.8%, 58.3%, 35.4% and 37.5%, respectively. The cumulative mortality in SB2 and SB3 groups was significantly lower than that in SB0 group (*P*<0.05). The cumulative mortality decreased linearly at 3 h (*P*< 0.05). By quadratic regression analysis, we found that the mortality from hypoxic stress was minimal when the supplemental dose of SB was 1.62g/kg (**Figure 1**).

**Table 8:** Cumulative mortality rate (%) of largemouth bass under hypoxic stress at the end of the 56-day test. ^a,b,c^ Bars without common superscripts indicate significant differences (*P*<0.05).

Item^3^	Dietary SB^1^ supplementation				SEM^2^	*P*-value	Polynomial contrasts	
	SB0	SB1	SB2	SB3			Linear	Quadratic
1 h	25.00	25.00	14.58	8.33	7.065	0.115	0.027	0.549
2 h	50.00^a^	39.58^ab^	22.92^b^	18.75^b^	8.715	0.023	0.004	0.626
3 h	68.75^c^	58.33^ab^	35.42^c^	37.50^bc^	9.081	0.016	0.004	0.359

^1^SB, sodium butyrate. SB0, basal diet; SB1, basal diet supplemented with 0.5 g/kg of sodium butyrate; SB2, basal diet supplemented with 1.0 g/kg of sodium butyrate; SB3, basal diet supplemented with 2.0 g/kg of sodium butyrate.

^2^CMR, cumulative mortality rate (%);

^3^1h, 2h, and 3h represent 1 hour, 2 hours, and 3 hours of hypoxic stress, respectively.

## 4 Discussion

In this study, the effects of SB on growth performance, serum biochemistry, immuno-antioxidant and hypoxic stress of largemouth bass were investigated. In this study, SB was found to improve the growth performance of largemouth bass, which was confirmed by analyzing terminal weight gain, weight gain rate, feed efficiency and protein deposition rate. Studies have shown that dietary sodium butyrate improves the growth of fish such as Nile tilapia (47), turbot (*Scophthalmus maximus*) (31), Asian sea bass (*Lates calcarifer*) (48), carp (*Cyprinus carpio*) (23), tilapia (28), crucian carp (*Carassius auratus tripl*) (49), and sea bream (*Sparus aurata*) (50). SB has been shown to improve fish growth and intestinal health. First, butyrate promotes the growth and development of intestinal villi and increases the contact area with nutrients (23). Secondly, butyrate can provide essential amino acids and vitamins for fish growth, or increase the activity of digestive enzymes and improve the intestinal flora to promote the digestion and absorption of nutrients (50, 51). Finally, butyrate can improve cellular immunity and antioxidant capacity to improve intestinal health (52). The present study found that dietary SB supplementation increased the expression of growth hormone (*GH*) and insulin-like growth factor 1 (*IGF-1*) in the liver. Growth hormone (*GH*), mediated by insulin-like growth factor (*IGF*), plays an important role in the regulation of fish growth, and growth hormone levels affect the growth rate of fish (53, 54). Similarly, dietary granule sodium butyrate was found to significantly increase *GH* and *IGF-1* mRNA expression in Nile tilapia (55). However, the results of this study are inconsistent with the literature, as no differences in SB on body weight gain were observed in Atlantic salmon [0.5-2%, (56)], rainbow trout [0.5-2%, (57)], red hybrid tilapia [200-2000 mg/kg, (58)] and African catfish trout [200-2 000 mg/kg, (52)]. Feed supplementation with low doses (0.1-0.2%) of butyric acid promoted fish growth, and feeding 0.3% sodium butyrate to juvenile grass carp had no significant effect on their growth performance (59). The study found 0.2% to 0.3% sodium butyrate to be the appropriate dose for European seabass (60). The study found that there is a dose-effect relationship between SB and fish growth (32, 59), with low doses of butyric acid promoting fish growth, and 0.3% sodium butyrate having no significant effect on the growth performance of juvenile grass carp. Interestingly, in this experiment, the addition of different doses of SB (max. 2%) all promoted the growth of largemouth bass, which is consistent with the conclusion of a previous study in juvenile yellow catfish, and the reason for this may be related to the dosage of SB and the species of fish (32). Differential responses to SB addition on growth performance may be related to diet composition, intestinal maturation status, and SB dose (61).

Short-chain fatty acids play an important role in nutrient metabolism. Butyrate can enhance the ability of the intestine to produce SCFAs by increasing the *Lactobacillus* flora in the intestine, and acetate produced in the intestine can be converted into acetyl-coA, which can be used as high-energy substrates in the citric acid cycle or for lipid synthesis (29, 62, 63). This study examined the effects of dietary supplementation with SB to improve nutrient retention in juvenile largemouth bass. It has been reported that crude protein and ash levels of juvenile yellow catfish supplemented with 1 000 mg/kg SB diet were significantly increased, and crude lipid levels of juvenile yellow catfish supplemented with 2 000 mg/kg SB diet were significantly increased (32). A diet supplemented with sodium butyrate led to an increase in crude lipid and crude protein levels in Pacific white shrimp, *Litopenaeus vannamei* (64). However, dietary sodium butyrate supplementation did not significantly affect crude protein, crude fat, crude ash, and water content in rainbow trout (65), golden trout (*Trachinotus ovatus*) (33), and grass carp (59). Therefore, the differences in aquatic animal species may also be one of the reasons for the differences in results.

Multiple components of serum are dynamically balanced in response to environmental and dietary changes, and serum biochemical indices reflect the nutritional metabolism and health status of animals (66). Reduced serum urea nitrogen concentration is associated with nitrogen utilization efficiency (67, 68), and in the present study, dietary supplementation with SB significantly reduced serum urea concentration, suggesting that improved nitrogen utilization by SB was confirmed. Consistent with the results of the present study, SB reduced serum urea nitrogen concentration in pigs (69, 70). GLU is a major source of energy for body tissues and cells, and serum levels of GLU are closely related to digestion and absorption (71). In the present study, serum GLU levels were found to be significantly higher in largemouth bass fed SB. Ebrahimi et al. (2017a) reported a trend of elevated plasma glucose in red hybrid tilapia treated with dietary butyrate as compared to the control group. Similarly, feeding 5.0 g/kg butyrate was found to increase GLU levels in triploid carps compared to controls (72). In addition, butyrate increased the transcript levels of genes related to energy metabolism and fatty acid metabolism in the human colon (73). Previous studies have shown that butyrate inhibits fat accumulation and reduces obesity induced by a high-fat diet in mice (74, 75). In this study, SB reduced serum TG, CHO, HDL, and LDL levels in largemouth bass. Studies in large yellow croaker have shown that appropriate amounts of butyrate can regulate the expression of genes involved in lipid synthesis and lipid oxidation, thereby reducing lipid content in large yellow croaker (76). Oral administration of short-chain fatty acids, including butyrate, may reduce lipid deposition by decreasing lipogenesis and enhancing lipolysis in different tissues (77). Fang et al. (78) and Ullah et al. (82) found that SB reduced the HSI, which is consistent with the results of the present study, and may be related to the reduction of lipid content in the livers of largemouth bass by sodium butyrate. As in the present study, SB reduced TG content in the liver of largemouth bass (79), and additional SB supplementation reduced TG in grass carp on a high-fat diet (80). However, the underlying mechanism of sodium butyrate’s effects on lipid metabolism in aquatic animals remains to be elucidated.

ROS originate mainly from energy metabolism, and ROS production is associated with homeostasis of mitochondrial activity and cellular redox reactions (81). Antioxidant enzymes are crucial for scavenging free radicals and preventing the production of reactive oxygen species (39, 82–84). Lipid peroxidation is a common result of oxidative stress, leading to the production of the toxic by-product malondialdehyde (MDA), which can cause mutations and atherosclerosis (85). Supplementation of organic acids, such as SB, has been shown to improve the antioxidant capacity and protect the health of aquatic animals (86). T-AOC is an indicator for assessing the overall efficacy of the antioxidant defense system in an organism, which encompasses the performance of both enzymatic and non-enzymatic systems in the fight against free radical metabolism and compensatory capacity (87). This study showed that total antioxidant capacity (T-AOC) level was significantly increased and MDA level was significantly decreased in largemouth bass fed SB diet. SB inhibited glycogen synthase kinase 3β (GSK-3β) and increased the activity of nuclear factor erythroid 2-related factor 2 (Nrf2), thereby increasing the expression of downstream antioxidant enzymes and reducing oxidative stress in cells (37). Similar to our study, under heat stress conditions, sodium butyrate supplementation improved the activity of antioxidant enzymes (SOD, CAT, and GSH-Px) in Nile tilapia while reducing the MDA content, effectively improving the antioxidant capacity of the fish (39). The findings in yellow catfish were the same as the results of this experiment, and SB up-regulated the expression levels of SOD, CAT, and GSH-Px mRNAs under ammonia stress, which improved the resistance (41). Overall, the results suggest that SB has a positive effect on activating the antioxidant defense mechanism of largemouth bass, enhancing the activity of antioxidant enzymes, and improving their stress resistance.

Stimulation of the host immune response enhances the protection of fish against bacterial and viral diseases; therefore, this experiment evaluated the changes of SB on the expression levels of immune-related genes in the liver of largemouth bass. Transforming growth factor beta (*TGF-β*) has been identified as a chemokine that can trigger inflammation; it is associated with the malignancy of many cancers and the defective response to growth inhibition, and it has a significant impact on immunosuppression (88). SB can inhibit cancer cell invasion and metastasis by inhibiting transforming growth factor-β1 (*TGF-β*1) (89). Butyrate blocked the signal transduction of *TGF-β*1 by inhibiting the expression of *TGF-β*1 mRNA (90). The results of the present study showed that SB decreased *TGF-β*1 expression water, indicating that SB alleviated the immunosuppressive effect of *TGF-β*1 on largemouth bass. It is interesting that opposite results of SB were observed in yellow drum (*Nibea albiflora*) (91) and grass carp (51), and the specific reasons need to be further studied. When an inflammatory response occurs, cells reduce damage from the inflammatory response by upregulating mRNA levels of anti-inflammatory factors (e.g., *IL-10*) or downregulating pro-inflammatory factors (e.g., *TNF-α, IL-8, IL-1β*) (92). Toll-like receptors (*TLR*) recognize and bind pathogen-associated molecular patterns (*PAMP*), and upon recognition, *TLR* interacts with myeloid differentiation factor 88 (*MyD88*) to activate *NF-κB* and induce the production of cytosolic pro-inflammatory factors (93). Nuclear factor kappa B (nuclear factor kappa B, *NF-κB*) proteins are protein complexes that control transcription of DNA, cytokine production and cell survival (36, 94). Toll-like receptor 22 (*TLR22*) and *MyD88* have been shown to play important roles in the innate immune response of grass carp (95) and weanling piglets (70). Butyric acid has been found to inhibit the release of *NF-κB* and prevent its translocation from the cytoplasm to the nucleus, ultimately inhibiting the expression of pro-inflammatory cytokines (96). In this study, dietary SB downregulated the expression levels of the pro-inflammatory factors *TNF-α, IL-1β, IL-8*, and *TLR22, MYD88* mRNA, with 1% supplementation with SB showing the best effect. The results showed that dietary sodium butyrate could effectively improve the immunity of largemouth bass and could down-regulate the mRNA expression level of pro-inflammatory cytokines regulated by the *TLR22-MyD88-NF-κB* signaling pathway. The results of previous studies were consistent with the present study in that SB inhibited the *NF-κB p65* signaling pathway, improved 2,4,6-trinitrobenzene sulfonic acid-induced intestinal inflammation in mice, and maintained intestinal integrity (97). This study suggests that this anti-inflammatory potential of butyrate may be related to the inhibition of the *NF-κB-P65* pathway. Another study in grass carp showed that cytokine regulation might be involved in the *NF-kB-p65* signaling pathway and confirmed that SB supplementation improved resistance to enteritis in grass carp (98). In conclusion, butyrate reduces inflammatory damage by activating *TLR*22-*MyD*88 signaling pathway to reduce the expression of inflammatory factors and enhance resistance to inflammatory responses.

Fish stressors include fish transport, high or low body temperature, hypoxic conditions, high or low salinity, poor nutrition, and various types of contaminants. These environmental changes can disrupt the dynamic balance of ROS in fish and thus produce a stress response that affects the normal morphology and physiology of fish (99, 100). Effective levels of dissolved oxygen (DO) are required for aerobic fish metabolism, and in intensive, high-density culture environments, DO in water is a major limiting factor in fish farming (101). If a portion of the fish’s energy is used to respond to stressors, less energy is available for other biological functions, including immunity (102, 103). Hypoxia may cause damage to the immune system of fish and reduce resistance to pathogenic infections (104). Hypoxic stress reduces antioxidant enzyme activity and increases malondialdehyde levels (101). In previous studies, it has been demonstrated that dietary supplementation with short-chain fatty acids (e.g., sodium propionate) can increase the expression levels of antioxidant enzyme genes in carp (105). Furthermore, there have been numerous studies evaluating the effect of SB addition to the diet on the antioxidant activity of different fish species (40, 106–108). In this study, placing largemouth bass under low oxygen emergency conditions, dietary supplementation with SB was effective in reducing the cumulative number of fish mortality, with 1.0 g/kg SB supplementation having the lowest cumulative mortality, which is consistent with the analysis of MDA, indicating that fish in the 1.0 g/kg supplementation group had the strongest antioxidant capacity and showed the best resistance to low oxygen stress. Similar to our study, the addition of SB to the diet up-regulated the expression levels of antioxidant enzymes (e.g., SOD, CAT, and GSH-Px) in rainbow trout (40) and increased activities of antioxidant enzymes (T-AOC, SOD, and GSH-Px) and reduced MDA levels were also found in the intestine of grass carp juvenile (109). SB decreased the expression of tumor suppressor gene *P53* and pro-apoptotic gene Bax and inhibited cell apoptosis under ammonia stress (41). In conclusion, dietary supplementation of SB can effectively reduce the cumulative mortality of largemouth bass under anoxic stress. The reason may be that SB can increase the activity of antioxidant enzymes and reduce inflammatory factor production and inhibit cell damage and apoptosis by decreasing *TLR22*, *MyD88*, and *TGF-β1* expression levels, thus reducing mortality under hypoxic stress conditions, but the mechanism of effect still needs to be further explored.

## 5 Conclusions

The data obtained in this study detailed that SB could better improve the growth performance of largemouth bass by increasing antioxidant enzyme activity, while activating *TLR*22-*MyD*88 signaling pathway and down-regulating inflammatory factor expression levels. In the hypoxic stress test, SB significantly increased the survival rate and showed good antioxidant capacity.

## Data availability statement

The original contributions presented in the study are included in the article/supplementary files. Further inquiries can be directed to the corresponding author.

## Ethics statement

The animal study was approved by Animal Care and Use Committee of the Guangdong Academy of Agricultural Sciences. The study was conducted in accordance with the local legislation and institutional requirements.

## Author contributions

DH: Data curation, Methodology, Writing – original draft. ML: Investigation, Writing – original draft. PL: Methodology, Supervision, Writing – review & editing. BC: Formal Analysis, Project administration, Writing – review & editing. WH: Supervision, Writing – review & editing. HG: Validation, Writing – review & editing. JC: Methodology, Resources, Supervision, Writing – review & editing. HZ: Formal Analysis, Methodology, Supervision, Writing – review & editing.
